# Non-monotonic changes in critical solidification rates for stability of liquid-solid interfaces with static magnetic fields

**DOI:** 10.1038/srep20598

**Published:** 2016-02-05

**Authors:** W. L. Ren, Y. F. Fan, J. W. Feng, Y. B. Zhong, J. B. Yu, Z. M. Ren, P. K. Liaw

**Affiliations:** 1 State Key Laboratory of Advanced Special Steel, College of Materials Science and Engineering, Shanghai University, Shanghai 200072, PR China; 2 Department of Materials Science and Engineering, The University of Tennessee, Knoxville, TN37996, USA

## Abstract

We report the magnetic field dependence of the critical solidification rate for the stability of liquid-solid interfaces. For a certain temperature gradient, the critical solidification rate first increases, then decreases, and subsequently increases with increasing magnetic field. The effect of the magnetic field on the critical solidification rate is more pronounced at low than at high temperature gradients. The numerical simulations show that the magnetic-field dependent changes of convection velocity and contour at the interface agree with the experimental results. The convection velocity first increases, then decreases, and finally increases again with increasing the magnetic field intensity. The variation of the convection contour at the interface first decreases, then increases slightly, and finally increases remarkably with increasing the magnetic field intensity. Thermoelectromagnetic convection (TEMC) plays the role of micro-stirring the melt and is responsible for the increase of interface stability within the initially increasing range of magnetic field intensity. The weak and significant extents of the magneto-hydrodynamic damping (MHD)-dependent solute build-up at the interface front result, respectively, in the gradual decrease and increase of interfacial stability with increasing the magnetic field intensity. The variation of the liquid-side concentration at the liquid-solid interface with the magnetic field supports the proposed mechanism.

The stability of a liquid-solid interface during solidification and the subsequent reorganization of the interface into a periodic array of cells or dendrites are of great interest. The criterion for interface stability was first proposed by Tiller *et al.*^1^ through considering the temperature gradient and solidification rate at the growth front. Mullins *et al.* extended the criterion to include interfacial energy^2^. Since then, the effects of various factors on the interface stability have been investigated, including the effects of magnetic fields^3^, natural convection^4^,^5^, flow owing to the density change during solidification^6^, the thermocapillary force at the free boundary^7^, forced flows^8^,^9^,^10^, composition-generated elastic stress^11^, and crystal orientation^12^.

The effects of magnetic fields on alloy and semiconductor solidification have generated significant interest, because magnetic fields can improve the physical properties of materials. Early studies explored classical magneto-hydrodynamic damping (MHD) effects, such as motion braking, convection stabilization, and free surface shaping^13^,^14^. In the motion braking effect, the interaction of the melt convection with a static magnetic field generates a force that opposes the flow motion in the solidifying melt. The braking Lorentz force is expressed as:


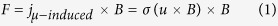


where *σ* is the electrical conductivity of the liquid, *B* is the strength of the applied magnetic field, and *u* is the flow velocity. This formula has been widely used in the metallurgical industry for suppressing melt turbulences and flow instabilities^13^,^14^. Later studies accounted for the thermoelectromagnetic convection (TEMC) effect of the magnetic field^15^,^16^,^17^,^18^. The flow is induced by the interaction between the magnetic field and thermoelectric current owing to a thermoelectric power difference between the liquid and solid phases at the growth front. It can give rise to distorted cellular arrays, freckles, and striations^16^,^18^. However, the appropriately regulating the TEMC intensity could make the liquid-solid interface flat and the Si concentration along the interface uniform during the growth of Ge_1−x_Si_x_ crystal^17^. Khine *et al.* also pointed out that TEMC can cancel the composition-driven buoyant convection and then eliminate radial macro-segregation in the crystal^15^.

MHD and TEMC have competing roles during directional solidification. Khine *et al.* studied the magnitude of the meridional circulation. It first increases from zero to the maximum at the Hartmann number of ~10, and then decreases toward zero, because the MHD effect increases faster than the TEMC effect^15^. Li *et al.*^19^ reported different TEMC maxima on the scale of cells/dendrites and samples. The TEMC effect corresponds to a more intense magnetic field on a smaller scale.

Accordingly, the effect of a magnetic field on the liquid-solid interface stability has been reported^3^,^18^,^20^. Using linear stability analysis, Coriell *et al.*^3^ had theoretically investigated the MHD effect, showing how the critical concentration of convective instabilities could be increased. Li *et al.*^18^ primarily found that a strong magnetic field causes the instability of a planar interface. They proposed that the solute build-up in the diffusion boundary layer and the stress formation in the solid near the interface are responsible for the interface breakdown. The stress formation is due to the thermoelectric magnetic force in the solidified solid^20^.

In our previous work, we reported the changes in liquid-solid interface stability incurred by magnetic fields^21^,^22^. The interface stability increased, decreased, and then increased again, for magnetic field intensity in the 0–0.4 T range, 0.4–0.8 T range, and above 0.8 T, respectively. Interestingly, the interface with an appropriate magnetic field was more stable than that without the magnetic field, which was different from what has been reported in the literature^18^,^19^. Khine *et al.* predicted that some TEMC effects could cancel the composition-driven buoyant convection, causing the crystal to exhibit more homogenous radial macro-segregation^15^.

Extending the previous work, in the present research, we found that the critical solidification rate for the interface stability changes with the characteristic magnetic field intensity (0, 0.4, 0.8, and 4 T). The underlying mechanism was explored using melt-velocity simulations and solute-concentration experimental investigations near the interface along the growth direction. The TEMC effect in the interface liquid plays a micro-stirring role and, thus, improves the interface stability, resulting in the maximization of the critical solidification rate with increasing the magnetic field intensity. The MHD at the interface exerts two effects with increasing the magnetic field intensity: First, it induces the solute build-up, and results in the instability for moderate intensities of the magnetic field. Second, when the solute builds up to some extent at the growth front, the melt convection at the interface is induced, and stability is again improved. The solute concentration in the liquid at the interface melt supports this proposal. The novel mechanisms revealed in the present study extend and deepen our knowledge of the contribution of magnetic fields to directional solidification. The present research provides helpful guidance for applying magnetic fields to control solidification.

## Results

**Figure 1:**
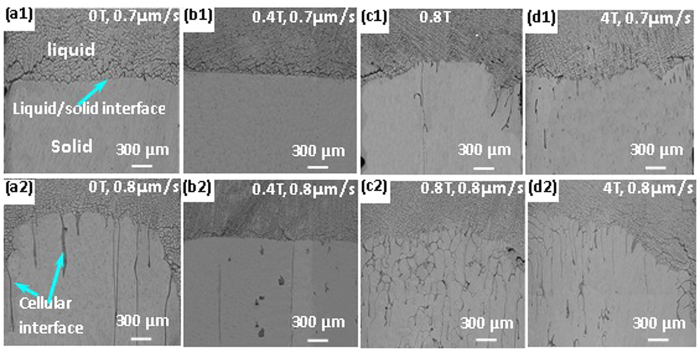
The morphology of the liquid-solid interface of the Al-0.85 wt.% Cu alloy at the solidification rates and different magnetic field intensities (The condition is indicated at the top right corner of each graph, G = 47.4 K/cm).

Based on the previous investigation^21^,^22^, the characteristic strength of the magnetic field and solidification rate were chosen for studying the interface stability. Figure 1 shows the effect of the magnetic field on the interface stability at the solidification rates of 0.7 and 0.8 μm/s, for the temperature gradient of 47.4 K/cm. The irregular lines in the solid mark the cellular interface. Thus, these lines indicate the extent of the interface instability. At 0.8 μm/s, the number of lines in the solid near the interface first decreases, then increases, and finally decreases again with increasing magnetic field intensity. This trend indicates that the interface stability first increases, then decreases, and finally increases again with increasing magnetic field intensity. The dependence of the interface stability on the magnetic field intensity agrees with what has been shown in refs 21 and 22. At 0.7 μm/s, the interface is stable for the magnetic field intensities of 0 and 0.4 T, while it is unstable for the magnetic field intensities of 0.8 and 4 T.

**Figure 2:**
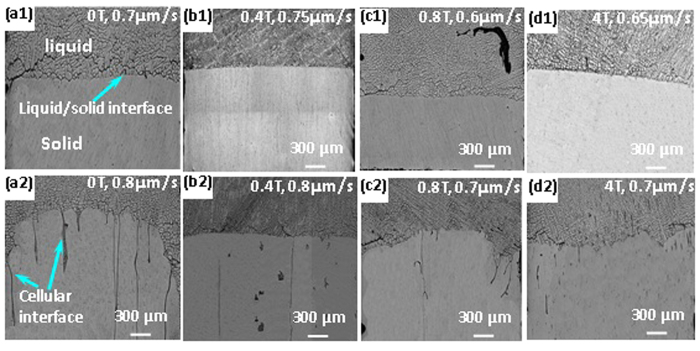
The morphology of the liquid-solid interface of the Al-0.85 wt.% Cu alloy at critical solidification rates for achieving stability at different magnetic field intensities (G = 47.4 K/cm).

To determine the critical solidification rate to achieve interface stability, the deviation of 0.05 μm/s was adopted for the solidification rates of 0.7 or 0.6 μm/s in different magnetic fields. Figure 2 shows the interface stability near the critical solidification rate, in the presence of different magnetic fields. The critical solidification rates for achieving the interface stability were 0.70, 0.75, 0.60, and 0.65 μm/s for the magnetic field intensities of 0, 0.4, 0.8, and 4 T, respectively, for the temperature gradient of 47.4 K/cm. The critical solidification rate first increased, then decreased, and then increased again with increasing the magnetic field intensity.

**Figure 3:**
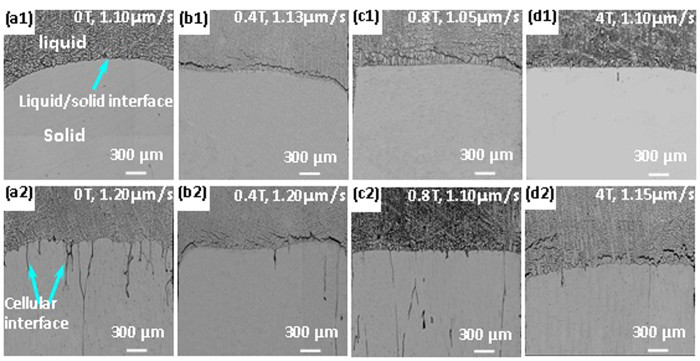
The morphology of the liquid-solid interface of the Al-0.85 wt.% Cu alloy at the critical solidification rates for achieving stability and for different magnetic field intensities (G = 81.5 K/cm).

To confirm the variation of the critical solidification rate with the magnetic field intensity, the effect of the magnetic field on the critical solidification rate was investigated for different temperature gradients. Figure 3 shows the interface stability for different magnetic field intensities, for the temperature gradient of 81.5 K/cm. The critical solidification rates for achieving the interface stability were 1.10, 1.13, 1.05, and 1.10 μm/s for the magnetic field intensities of 0, 0.4, 0.8, and 4 T, respectively, for the temperature gradient of 81.5 K/cm. The variation of the critical solidification rate with the magnetic field exhibited the same trend as that for the temperature gradient of 47.4 K/cm, i.e., as increasing the magnetic field intensity, the critical solidification rate first increased, then decreased, and increased again.

**Figure 4:**
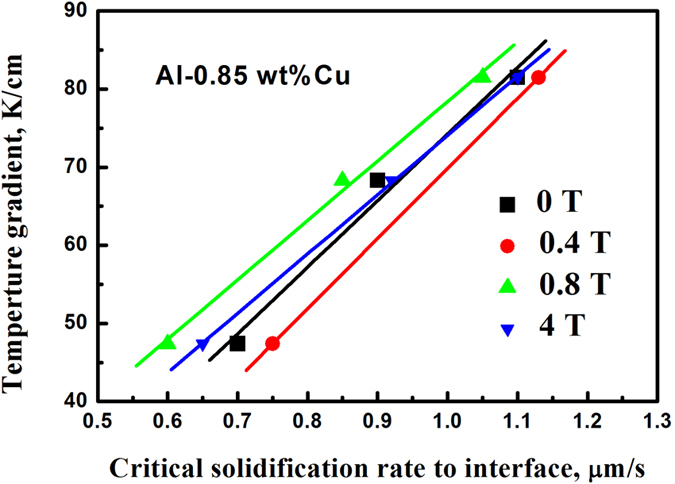
The dependence of the critical solidification rate for achieving the liquid-solid interface stability on the temperature gradient for the Al-0.85 wt.% Cu alloy in different magnetic field intensities.

Figure 4 summarizes the critical solidification rate for the interface stability and for the different magnetic field intensities and temperature gradients. It can be seen that, for a certain temperature gradient, the critical solidification rate first increases, then decreases, and then increases again with increasing the magnetic field intensity. For low temperature gradients, the range of critical solidification rates is wider than that for high temperature gradients. This trend indicates that the effect of the applied magnetic field becomes more pronounced for low temperature gradients.

## Discussion

The results show the critical solidification rate exhibits a non-monotonic change with magnetic field. As is known, the critical solidification rate represents the stability of liquid-solid interface during directional solidification. That is to say, the interface would get into an instable condition when the solidification rate is over the critical value. Thus, the effect of magnetic field on the critical solidification rate is achieved by its acting on the interface stability. Therefore, the below discussion is concentrated on the influence of magnetic field on the interface stability.

The interfacial stability is evaluated according to the following equation:


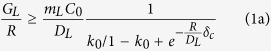


where *G*_*L*_ is the temperature gradient at the growth front, *D*_*L*_ is the solute diffusion coefficient in the melt, *R* is the solidification rate, *m*_*L*_ is the liquid’s slope, *C*_*0*_is the initial concentration of the solute, *k*_*0*_ is the equilibrium partition coefficient, and *δ*_*c*_ is the thickness of the solute-rich layer (SRL).

The magnetic field intensity in this study was not sufficiently large to affect the thermodynamic parameters *k*_*0*_ and *m*^18^, and the solidification parameters, *G*_*L*_, *R, D*_*L*_, and *C*_*0*_ were not modified by the applied magnetic field. Therefore, the SRL’s thickness, *δ*_*c*_, was affected by the convection in the melt. More intense convection yielded thinner SRL. The effect of the magnetic field on the solidification convection mainly involves two aspects: the MHD and TEMC. The MHD plays a braking role in the melt, and the TEMC induces a flow in the melt. These two effects compete. Khine *et al.* showed that the magnitude of the meridional circulation first increased from zero to a maximum and then decreased, because the MHD effect increased faster than the TEMC effect^15^. Li *et al.* reported that a large-scale maximum of the TEMC effect corresponded to the low magnetic field intensity^20^. As is known, strong convection induces thin SRL and low concentration at the liquid-solid interface. Accordingly, the melt convection and the liquid-side concentration at the interface under the different magnetic fields were investigated for exploring the mechanism underlying the non-monotonic changes in the interface stability with increasing the magnetic field intensity.

Based on the boundary and initial conditions, the governing equations described above were discretized and temporally integrated using a finite-element method. The important physical parameters that were used in the simulations are listed in Table 1 in Supplementary. In these calculations, the temperature gradient was 47.4 K/cm, the magnetic field intensities were 0, 0.4, 0.6, 0.8, 1, 2, 4, and 8 T, and the solidification rates were 0.5, 1.0, and 1.5 μm/s.

**Figure 5:**
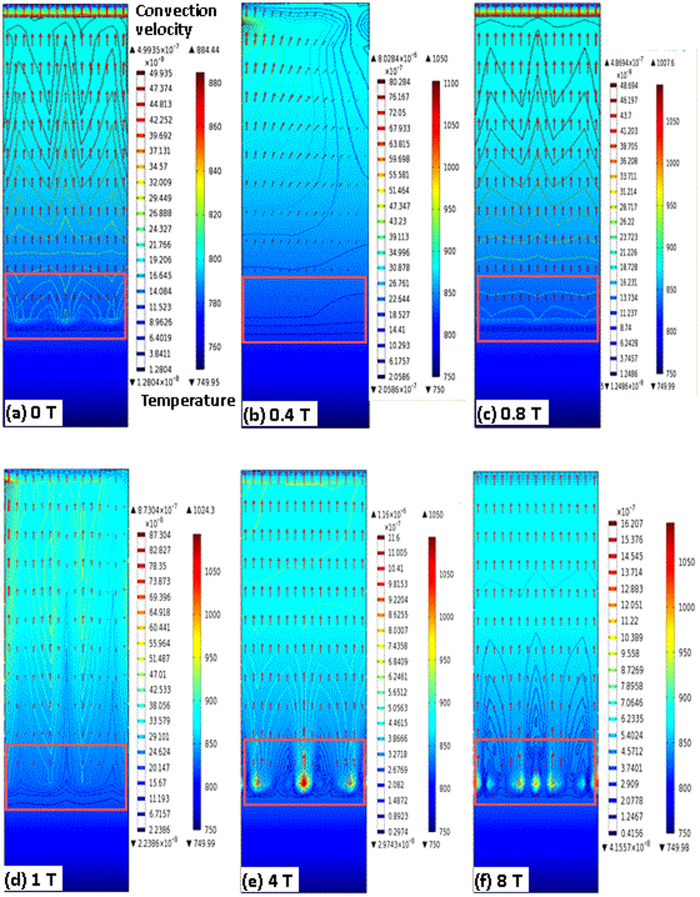
The convection-velocity field in the melt at the interface front and far away from the interface, at 1.0 μm/s, for the Al-0.85 wt.% Cu alloy, for the different magnetic field intensities. The lines indicate the velocity isograph, the arrows indicate the velocity magnitudes and directions, and the arrows are too sparse to show the upward ones. The red rectangle is shown near the liquid/solid interface to emphasize the convection structure at the growth front.

Figure 5 shows the temperature and velocity fields in the melt of Al-0.85 wt. % Cu at time t = 1,000 s, for the solidification rate of 1 μm/s, and for the different magnetic field intensities. The flow velocity and the velocity-contour maps change with the magnetic field intensity. For better comparison, the maximal and minimal velocity magnitudes for the different magnetic field intensities at the different solidification rates are shown in Fig. 1 in Supplementary. The velocity in the melt first increases, then decreases, and finally increases again with increasing magnetic field intensity. From Equation (7), the larger convection velocity in the melt would yield the thinner SRL and more stable interface. Therefore, this tendency of melt convection agrees with the interface stability as a function of the magnetic field intensity.

Let us examine the convection structure at the front of the liquid-solid interface in Fig. 5, indicated by the red rectangles. The convection contour at the interface first decreases, then slightly increases, and finally increases significantly. The TEMC

stems from the interaction of the magnetic field and the thermoelectric current at the interface, as shown in Fig. 2 in Supplementary. The TEMC plays a stirring role at the interface’s front in the radial direction, which decreases the convection contour as the magnetic field increases initially [Fig. 5(b)]. Khine *et al.* also proposed that the TEMC can cancel the composition-driven buoyant convection^15^. Therefore, the variation of the convection contour at the interface reaches a minimum due to the TEMC effect. The MHD effect on the melt convection strengthens the build-up of solutes at the growth front, causing the convection contour to increase [Fig. 5(c)]. However, when the solutes build up to a certain extent, the longitudinal convection becomes dominant at the interface front, because the convection cannot be suppressed by the MHD. The convection-governing Equation (4) of the *z-axis* component also indicates this trend, significantly affecting the convection contour’s dependence on the magnetic field intensity [Fig. 5(d–f)]. Therefore, the TEMC effect is responsible for the weak variation of the convection contour at the interface, and the weak and strong MHD effects result, respectively, in the weak and strong increases in the variation of the convection contour with increasing magnetic field intensity.

**Figure 6:**
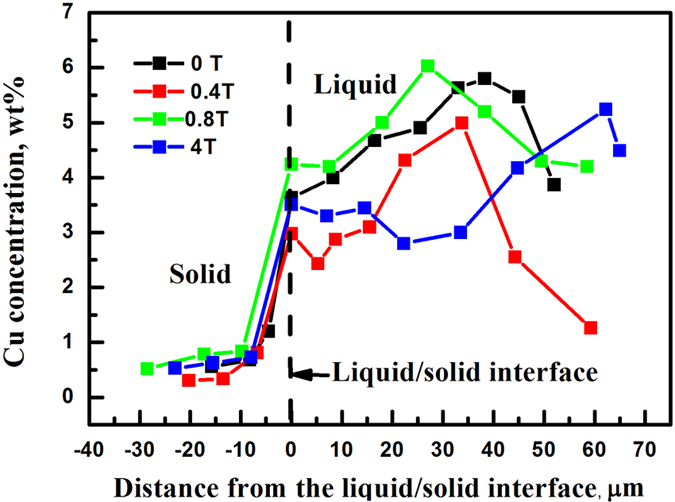
The longitudinal distribution of the solute Cu concentration near the liquid- solid interface for the Al-0.85 wt.% Cu alloy directionally solidified at 0.8 μm/s and 47.4 K/cm, for the different magnetic field intensities. Each dot represents the average value of the line scanning of energy-disperse spectroscopy (EDS) in Fig. 1 in Supplementary.

The effects of magnetic field on the melt convection finally embody the liquid concentration at the interface. Thus, we investigated the magnetic field dependence of the liquid concentration near the interface. Figure 6 shows the liquid concentration of the Al-0.85 wt.% Cu at the interface, for the solidification rate of 0.8 μm/s and temperature gradient of 47.4 K/cm, for the different magnetic field intensities. The concentration at the interface is less than that at the far melt because of the solute-back diffusion in the solid. The concentration at the interface first decreases, then increases, and finally decreases again with increasing the magnetic field intensity. This trend indicates that the melt-convection velocity first increases, then decreases, and finally increases again with increasing magnetic field intensity, which agrees with the above simulation results.

**Figure 7:**
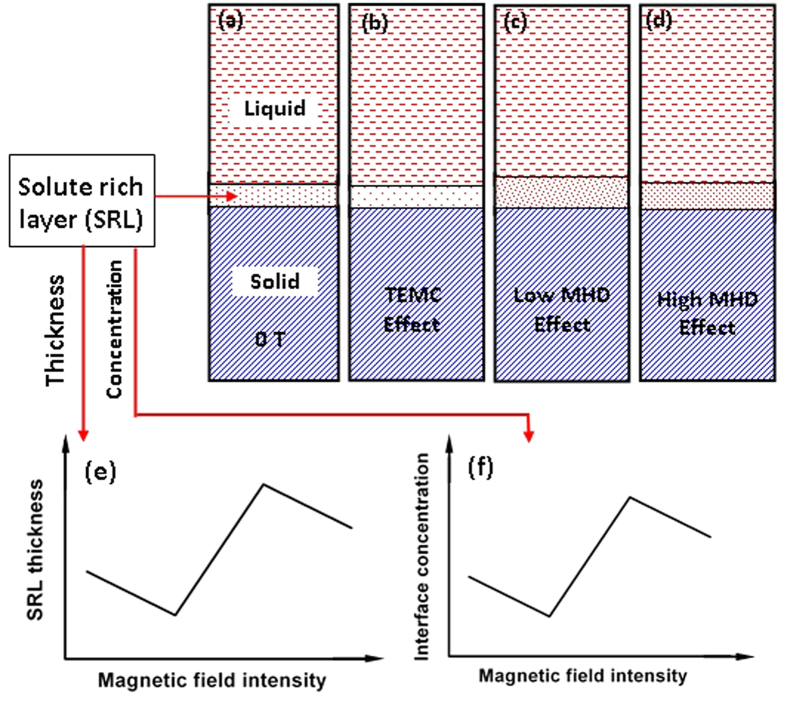
The schematic illustration of the magnetic-field dependent change of the solute-rich layer’s (SRL’s) thickness and interface concentration.

Therefore, the convention velocity and the structure, owing to the competition between the MHD and TEMC effects, modify the liquid concentration at the liquid/solid interface as well as the SRL’s thickness, as shown in Fig. 7. The TEMC in the liquid can result in a lower concentration at the interface and a thinner SRL [Fig. 7(b)]. The MHD effect will strengthen the solute’s build-up at the interface front. Accordingly, the SRL’s thickness increases [Fig. 7(c)]. However, when the solute builds up to a certain extent, the longitudinal convection velocity will increase, and the concentration at the interface and the SRL’s thickness will decrease again [Fig. 7(d)]. The changes in the SRL’s thickness and the interface concentration with increasing the magnetic field intensity are indicated in Fig. 7(e,f), respectively. The concentration at the growth interface further affects the interface stability as a function of the magnetic field intensity. And the change of interface stability ultimately reflects a variation in the critical solidification rate.

## Conclusions

We investigated the dependence of the critical solidification rate for achieving liquid-solid interface stability on the axial static magnetic field under different temperature gradients, and explored the associated mechanisms. The following conclusions can be drawn:

1. At a certain temperature gradient, the critical solidification rate first increases, then decreases, and then increases again with increasing the magnetic field intensity. For low temperature gradients, the effect of the magnetic field on the critical solidification rate is more pronounced than that for high temperature gradients.
2. The influence of magnetic field on the critical solidification rate is achieved through its acting on the liquid-solid stability. The simulation shows that the change in the convection velocity and contour at the interface with the magnetic field agrees with the trends that the interface stability varies with the magnetic field. The thermoelectromagnetic convection (TEMC) and the low and high magneto- hydrodynamic damping (MHD) effects play main roles in the different ranges of the increasing magnetic field intensity.
3. The change in the liquid-side concentration at the liquid-solid interface with the magnetic field further supports the experimental and simulation-based investigations. The TEMC’s micro-stirring role is responsible for a weaker build-up of the concentration at the interface, and enhances the interface stability as the magnetic field intensity increases initially. The different extents of the solute build-up at the interface front, induced by the MHD, result in an initial decrease and then increase of the interface stability with increasing the magnetic field intensity.

## Methods

An Al-0.85Cu (weight percent, wt.%) alloy used in this study was prepared with high-purity Al and Cu (99.99 wt.%) in an induction furnace. The raw alloys were placed in a high-purity graphite crucible and heated to 900 °C. Then, a glass tube with an inner diameter of ~3.5 mm was used to suck the melt for obtaining the samples. The 3.5-mm-diameter and 200-mm-long samples were enwrapped in a high-purity corundum tube for the directional solidification. The experimental apparatus for directional solidification in high-intensity magnetic fields was described in ref. 21. It mainly consisted of a static superconductor magnet, Bridgman-Stockbarger type furnace, drawing system, and temperature controller. The magnet produced a vertically oriented static magnetic field with an adjustable intensity of 14 T. A water-cooled cylinder containing liquid Ga-In-Sn metal was used for cooling down the specimen.

The liquid-solid interface was adjusted to the center plane of the magnet, at which the magnetic field intensity was homogenous. The samples were directionally solidified in the presence of various magnetic field intensities (0, 0.4, 0.8, and 4 T) and temperature gradients (47.4, 68.3, and 81.5 K/cm), near the solidification rates that were critical for the interface instability. To observe the morphology of the liquid-solid interface during the crystal growth, quenching experiments were conducted by quickly withdrawing the specimen into the liquid-metal cylinder. The longitudinal microstructures were examined in the etched condition, using an optical microscope. The longitudinal distribution of the solute Cu concentration near the interface was measured for the samples that were directionally solidified at the rate of 0.8 μm/s and under a temperature gradient of 47.4 K/cm, for different magnetic field intensities. The measurements were conducted, employing the line-scanning of energy-disperse spectroscopy (EDS) with a scanning electron microscope, which is described in Fig. 3 in Supplementary.

**Figure 8:**
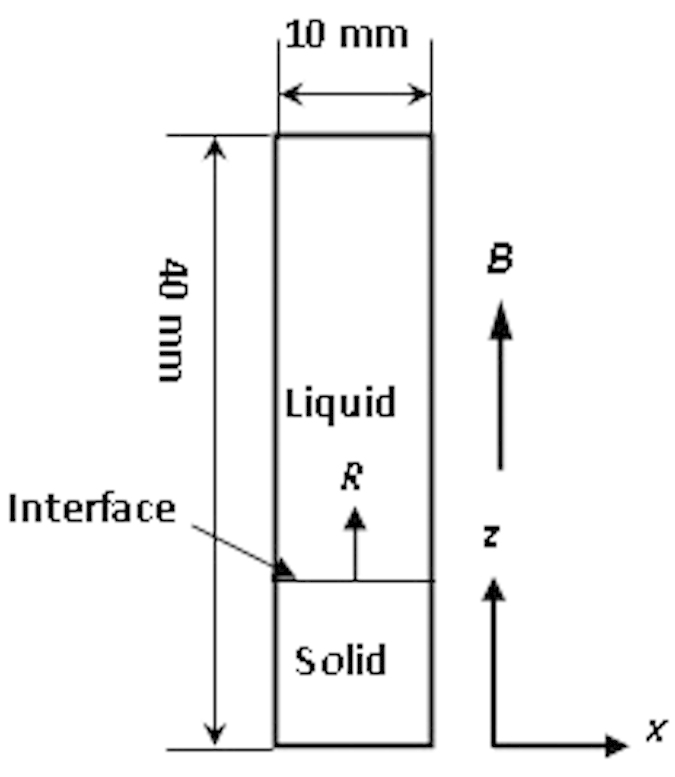
The schematic diagram and geometric definitions for the vertical Bridgman growth configuration. *B* is the magnetic field; *R* is the solidification rate.

In order to clarify the change of the liquid-solid interface with magnetic field from the convection aspect, the simulation calculation was performed. The model Bridgman system is presented in Fig. 8. A 10-mm-diameter and 40-mm-tall melt cylinder was chosen. The initial temperature of the melt was 1,050 K, and the alloy was Al-0.85 wt.% Cu.

The following assumptions were made:

1. Only the solid and liquid phases are present, i.e., no pores are formed.
2. The liquid is Newtonian and incompressible, and the flow is laminar.
3. The solid and liquid phases have the same thermal properties and densities.
4. There is no diffusion of solutes in the solid phase.
5. The thermal properties are constant, which allows the use of the Boussinesq approximation. Hence, the density is constant except in the body-force term of the momentum equation.
6. The solid is stationary. The liquid and solid concentrations at the interface are in the local equilibrium.

With these assumptions, the governing equations for the transport processes in the melt during crystal growth can be described by the conservation laws for the mass, the momentum, and the energy, as follows:


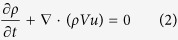


The *x-*axis component:


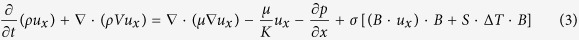


The z-axis component:


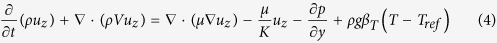



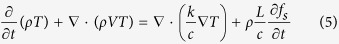


In the above equations, 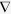 is the gradient operator, *V* is the drawing speed, *u* is the velocity, *ρ* is the melt density, *u*_*x*_ is the *x*-axis velocity, *u*_*z*_ is the *z*-axis velocity, *t* is the time, *μ* is the viscosity, *p* is the pressure, *g* is the gravity acceleration, *β* is the thermal expansion coefficient, *T*_*ref*_ is the reference temperature, *k* is the thermal conductivity, *c* is the specific heat, *L* is the latent heat, *f*_*s*_ is the volume fraction of the solid, *K* is the permeability, 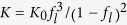, 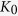 is the permeability constant, *σ* is the electrical conductivity, *S* is the thermoelectric power, *B* is the magnetic field intensity, 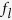 is the liquid’s volume fraction, *f*_*s*_ is expressed as 
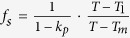
, *k*_*p*_ is the equilibrium partition coefficient, *T*_*l*_ is the liquid’s temperature, and *T*_*m*_ is the melt temperature of a pure alloy.

The temperature-concentration relation is:


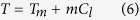


where *m* is the liquid’s slope, and *C*_*l*_ is the liquid’s concentration.

The boundary and initial conditions of the calculated domain are described in Fig. 4 in Supplementary.

## Additional Information

**How to cite this article**: Ren, W. L. *et al.* Non-monotonic changes in critical solidification rates for stability of liquid-solid interfaces with static magnetic fields. *Sci. Rep.* **6**, 20598; doi: 10.1038/srep20598 (2016).

## Supplementary Material

Supplementary Information
