# Supplementary material for: Non-monotonic changes in critical solidification rates for stability of liquid-solid interfaces with static magnetic fields
Source: Sci Rep. 2016 Feb 5;6:20598. doi: 10.1038/srep20598 (PMC4742885; doi:10.1038/srep20598)
Supplement: Supplementary Information [file srep20598-s1.doc]

**Non-monotonic changes in critical solidification rates for stability of liquid-solid interfaces with static magnetic fields**

W.L. Ren^*,a^, Y.F. Fan ^a^, J.W. Feng ^a^, Y.B. Zhong ^a^, J.B. Yu ^a^, Z.M. Ren ^a^, and P.K. Liaw ^b^

^a^ State Key Laboratory of Advanced Special Steel, College of Materials Science and Engineering, Shanghai University, Shanghai 200072, PR China

^b^ Department of Materials Science and Engineering, The University of Tennessee, Knoxville, TN37996, USA

*Corresponding author Tel.: +86-21-56336048; Fax: +86-21-56332939.

E-mail address: wlren@staff.shu.edu.cn, [yunboz@staff.shu.edu.cn](mailto:yunboz@staff.shu.edu.cn)

Fig. 1 The dependence of the total convection velocity of the melt on the magnetic field intensity of the Al-0.85 wt.% Cu alloy, at the different solidification rates: (a) 0.5 μm/s; (b) 1.0 μm/s; (c) 1.5 μm/s.


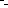

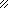

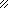

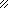


**B**

**Solid**

**Liquid**

**liLid**

**TEMC**

**I_TE_ TE**

**TEMFSolid**

**TEMFSolid**

Fig. 2 The schematic diagram of the thermoelectromagnetic convection (TEMC) micro-stirring. The difference of thermoelectric power between the liquid and solid induces the current (I_TE_) at the interface. The interaction between the horizontal component of I_TE_ and the magnetic field produces the thermoelectromagnetic force (TEMF), which vertically goes into the page in the right. The TEMF around the liquid-solid interface motivates the TEMC and exerts a micro-stirring effect.


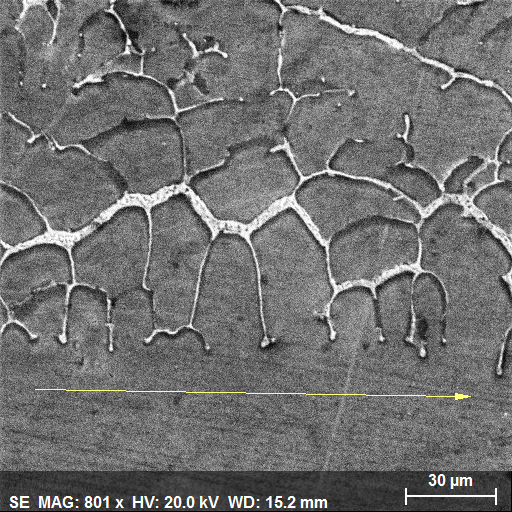


**EDS line-scanning trace**

Fig. 3 The schematic diagram of scanning methods for the solute measurement near the liquid-solid interface (the red lines indicate the trace of the line canning, the scanning length was 150 μm parallel to the interface, and the vertical scanning length at the liquid-solid interface covered 75 μm).

3

2

1

4

Fig. 4 The boundary and initial conditions of the calculated domain.

Figure 4 shows the boundary and initial conditions of the calculated domain. The velocity, temperature, and solute at the boundary 2 of the crystal/melt interface are:

$\frac{\partial p}{\partial z}=0$ (1)

$T=T_{t0}-G\cdot z$ (2)

$C=C_{0}+C_{0}\cdot k_{p}$ (3)

where *p* is the pressure, *z* is the displacement in the longitudinal direction, *T* is the temperature, *T_t0_* is the temperature at the top boundary and for the initial condition, *G* is the temperature gradient, *C* is the concentration, *C_0_* is the initial solute concentration, and *k_p_* is the equilibrium partition coefficient.

Table 1：Physical parameters used in the simulation for Al-0.85 wt.% Cu.

| Symbol | Value | Units |
| --- | --- | --- |
| $\rho$ | 2750 | $kg/m^{3}$ |
| $T_{f}$ | 934.2 | K |
| $C_{0}$ | 0.85 | wt% |
| $\mu$ | $2.43\times10^{-3}$ | $cm^{2}/s$ |
| D | $3.2\times10^{-5}$ | $cm^{2}/s$ |
| g | 980.0 | $cm^{2}/s$ |
| $\beta_{T}$ | $2.5\times10^{-5}$ | $K^{-1}$ |
| $k_{l}$ | 90 | $J/m\cdot s\cdot k$ |
| $k_{s}$ | 210 | $J/m\cdot s\cdot k$ |
| $L$ | $1.107\times10^{9}$ | $J/m^{3}$ |
| $k_{p}$ | 0.14 | – |
| $G_{L}$ | 47.4 | K/cm |
| $C_{p}$ | 900 | $J/kg\cdot K$ |
| *S* | 2.91×10^-6^ | *V · K^-1^* |
